# Set-Wise Differential Interaction between Copy Number Alterations and Gene Expressions of Lower-Grade Glioma Reveals Prognosis-Associated Pathways

**DOI:** 10.3390/e22121434

**Published:** 2020-12-18

**Authors:** Seong Beom Cho

**Affiliations:** Department of Biomedical Informatics, College of Medicine, Gachon University, Seongnam-Daero 1342, Korea; sbcho1749@gmail.com

**Keywords:** copy number alteration, gene expression, integrative analysis, Renyi’s relative entropy, the cancer gene atlas project, lower-grade glioma

## Abstract

The integrative analysis of copy number alteration (CNA) and gene expression (GE) is an essential part of cancer research considering the impact of CNAs on cancer progression and prognosis. In this research, an integrative analysis was performed with generalized differentially coexpressed gene sets (gdCoxS), which is a modification of dCoxS. In gdCoxS, set-wise interaction is measured using the correlation of sample-wise distances with Renyi’s relative entropy, which requires an estimation of sample density based on omics profiles. To capture correlations between the variables, multivariate density estimation with covariance was applied. In the simulation study, the power of gdCoxS outperformed dCoxS that did not use the correlations in the density estimation explicitly. In the analysis of the lower-grade glioma of the cancer genome atlas program (TCGA-LGG) data, the gdCoxS identified 577 pathway CNAs and GEs pairs that showed significant changes of interaction between the survival and non-survival group, while other benchmark methods detected lower numbers of such pathways. The biological implications of the significant pathways were well consistent with previous reports of the TCGA-LGG. Taken together, the gdCoxS is a useful method for an integrative analysis of CNAs and GEs.

## 1. Introduction

Copy number alteration (CNA) is a cytogenetic hallmark of cancer pathophysiology [1]. Due to the aberrant behavior of cancer cell proliferation and differentiation, genomic sequences can be amplified or deleted in cancer cells. The CNA can cause the abnormal expression of oncogenes or tumor suppressor genes. These abnormal expressions are related to cancer progression or poor prognosis [2,3,4,5,6]. For this reason, the identification of the copy number aberration has been a key issue in cancer research [7,8,9].

The array comparative genomic hybridization (aCGH) facilitated the discovery of the CNAs in cancer [7]. The paradigm of high-throughput technology, which is a massive parallelization of single experiments, was directly applied to the aCGH method. Consequently, researchers can obtain information about copy numbers on a genome-wide scale using the aCGH platform. Studies on many types of cancers revealed copy number anomalies in various genomic regions with the aCGH technology [8,9,10,11,12]. Recently, researchers have used a single nucleotide polymorphism (SNP) microarray platform for the detection of CNAs [13]. For the detection of CNAs, specific probes are inserted in the microarray platform. Several algorithms had been developed for analysis of the CNAs using the SNP microarray platform [14,15,16,17].

Although the microarray platforms enable the efficient screening of the CNAs, they give no information about gene expression (GE). For the identification of their impact on GE, they should be validated at the transcription level because the GEs of CNA loci can show no significant change [18]. To this end, the GE microarray or RNA sequencing platform can be used concurrently on the same samples that are applied to the CNA-detecting platform for accurate detection of the CNAs having an effect on transcription. The underlying assumption of the integrative analysis of the CNA and GE is straightforward: if the CNAs of genomic loci co-vary with the expression level of genes, it indicates that the genomic loci are likely to influence the GE.

The integrative analysis of the CNV and GE datasets has been focused on single gene-wise correlations or regression-based approaches that found significant relationships between CNA and GE, which are focused on identifying the coordinated variation between CNA and GE. To capture the variation, several computational methods were applied [19,20]. Lathi et al. reviewed and classified such methods into four categories, including two-step-, regression- and correlation-based approaches, and latent variable models [20]. The two-step approach consists of detecting CNA lesions and testing the association of the lesions and differential gene expressions. Regression- and correlation-based approaches are dependent on the corresponding statistical models that have been widely used in the data analysis, and some modifications of the original models are applied. Latent variable models are used to model the shared and independent signals between CNA and GE. This approach has an advantage in that it directly models the signal and noise, but has the disadvantage of high computation time.

In addition to the single gene-wise method, gene set approaches were also applied to the integrative analysis of CNA and GE. Menezes et al. used the global test to identify the relationship between single copy number alteration and corresponding gene set expression profiles [21]. By mapping neighbor expression probes to a single aCGH probe, they identified the CNAs that influenced the gene set expression profiles using the global test. The other gene set approach identified relationships between sets of CNAs and sets of expression values using canonical correlation analysis. Peng et al. applied the multivariate regression method for the set-wise analysis of CNAs and GEs [22]. To deal with the high dimensionality of genomic data, they used a regularization process. The canonical correlation analysis is a multivariate analysis method for detecting similarity between two variable sets. Lahti et al. used the canonical correlation method to determine a regional set of copy numbers and gene expression changes [23], which includes a probabilistic approach that is robust to small sample sizes. In another research, the elastic net approach was adopted to reduce the number of variables in the genomic data [24]. Similarly, selecting sparse subsets of variables of CCA instead of considering all combinations of genomic variables is proposed to consider high dimensional variables of genomic data [25].

In this research, the integrative analysis of CNA and gene expression is performed in terms of the gene set approach. The rationale for the set-wise analysis was to identify biological findings that were not detected by the single gene-wise analysis. Moreover, conditional changes in the similarity between CNAs and gene expressions are explicitly tested to identify whether a pair of CNAs and GEs is associated with the condition, which indicates that the CNAs and GEs are likely to be involved in the biology of the condition. For this purpose, the dCoxS method is modified to capture the variation between heterogenous omics data, especially for CNAs. The dCoxS was originally designed to detect interaction between a pair of GEs [26]. The interaction implies similarity between GEs, which is measured by the correlation between sample-wise distances in the GE matrices. For the identification of interactions between CNAs and GEs, dCoxS is able to be applied directly. However, if the CNAs data are in a segmented form, the dCoxS may not identify the combination effect of CNA loci in the determination of interaction because the dCoxS uses productive kernels for the estimation of sample-wise distances. Since the productive kernel computes the bandwidth parameters of the variables from the standard deviation of each variable, which can show monotonic variations in the segmented values of CNAs that represent only three statuses of gain, loss, and normal, the productive kernel may not be appropriate for the segmented CNA data. In this research, multivariate normal density estimation was applied, which integrates the correlation structure of the CNAs explicitly. Here, the modified method is named generalized dCoxS (gdCoxS), and it can analyze heterogenous omics datasets. The performance of the gdCoxS is tested using simulation data and lower-grade glioma of the cancer genome atlas program data.

## 2. Materials and Methods

### 2.1. Identification of Conditional Change of Interactions between Set-Wise CNAs and GEs

**Figure 1:**
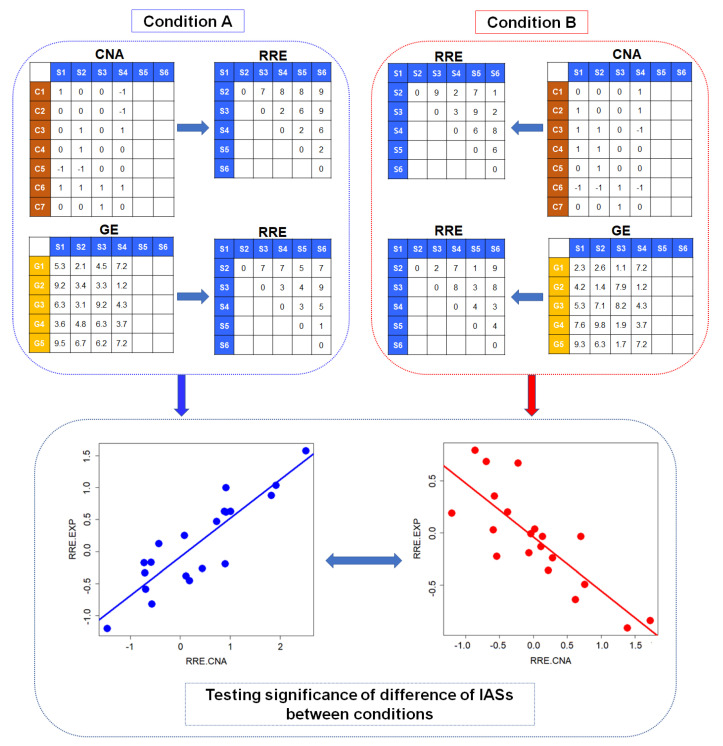
Overall analysis flow of generalized differentially coexpressed gene sets (gdCoxS). In each condition, copy number and gene expression matrices are converted to matrices of sample-wise distances that are measured by Renyi’s relative entropies. Then, interactions are determined by the computation of correlation coefficients of sample-wise distances from the copy number and gene expression matrix. CNAs: copy number alterations; GEs: gene expressions; IAS: interaction score; RREs: sample-wise distances with Renyi’s relative entropies.

The overview of analysis is illustrated in Figure 1. The dCoxS method was originally developed for detecting significant changes in the interaction of a pair of gene expression matrices between different conditions. In the dCoxS, conditional similarity between two gene set expression profiles was determined by the correlation of sample-wise distances in the expression profiles, which was defined as the interaction score (IAS).

For the estimation of sample-wise distances, Renyi’s relative entropy is estimated by the ratio of densities from two different samples. The densities were computed using the multivariate productive kernel that multiplies the single density values and bandwidth parameters obtained from standard deviations of the variables. The dCoxS performs well in the estimation of differential interaction between a set of gene expressions. However, when the method is applied to CNAs and gene expressions, the productive kernel may not represent the dynamics of CNA changes because it integrates no explicit correlation structure into the density estimation. The CNA status includes only three possible values, which are loss, neutral and gain, and these are frequently coded as −1, 0 and 1, respectively. Since the CNAs occur in a small portion of samples, it is likely that the density of the CNA matrix had small variations because combinations of the CNA status are not considered explicitly in the dCoxS. Thus, in this analysis, a multivariate normal density estimation that uses a covariance matrix representing combinations of the CNA status is adopted. The multivariate density function is:

(1) $$f(x)=\frac{1}{\sqrt{{\left(2\pi \right)}^{p/2}{\left|\Sigma \right|}^{1/2}}}e^{-{\left(x-\mu \right)}^{\prime }\Sigma^{-1}(x-\mu )/2}$$

where *n* and *p* represent the number of samples and variables. The *µ* is a mean vector of CNA or GE profiles, and ${\hat{\Sigma }}^{1/2}$ is the square root of the estimated covariance matrix. In practice, *n* was the number of samples and *d* was the number of CNAs or GEs in a pathway. The corpcor R package was used for the shrinkage estimation of the covariance matrix and its inverse form [27] to handle the computation of high-dimensional matrices that are frequently possible with various types of genomics data (*n* < *p*). 

For each corresponding copy number and expression matrix, sample-wise distances were measured with Renyi’s quadratic divergence.

(2) $$D_{2}(P||Q)=\log\frac{{\hat{f}}_{h}(S_{i})}{{\hat{f}}_{h}(S_{j})}$$

In Equation (2), *D*_2_*(P*||*Q)* represents Renyi’s quadratic diversity [26]. The *S_i_* and *S_j_* indicate different samples. The ${\hat{f}}_{h}(S_{i})$ and ${\hat{f}}_{h}(S_{j})$ are the probabilistic densities of the samples *S_i_* and *S_j_*. Therefore, the higher divergence implies that two samples are more distant from each other. 

Using the Renyi’s diversity, set-wise CNA and expression matrices were transformed to sample-wise distance matrices. The upper trigonal members of the sample-wise distance matrices were used for the computation of the IAS. The IAS was obtained through the correlation coefficient between the upper trigonal members of the sample-wise distance matrices.

(3) $$IAS=\frac{\sum {}_{i<j}(RE^{C}-\bar{RE^{C}})(RE^{G}-\bar{RE^{G}})}{\sqrt{\sum {}_{i<j}{\left(RE^{C}-\bar{RE^{C}}\right)}^{2}}\sqrt{\sum {}_{i<j}{\left(RE^{G}-\bar{RE^{G}}\right)}^{2}}}$$

In Equation (3), *RE^C^* and *RE^G^* are the sample-wise distance (relative entropy) matrices of the CNAs and GEs, respectively. After the IASs were determined in each condition, the significance of the IAS and the differences in the IAS between conditions were tested non-parametrically (Supplementary Methods). 

### 2.2. Simulation Analysis

Since the IAS is used for determining the similarity between set-wise CNA and gene expression matrices, unlike the original application, a simulation study tests whether the IAS reflects the similarity between CNAs and GEs.

First, a CNA matrix was generated using binomial distribution. In general, CNA occurs in a small proportion of samples. Neutral status was therefore set to the predefined proportion of total samples. Then, gain (+1) or loss (−1) status was assigned to the rest of the samples using binomial distribution with number of trials = 1 and probability = 0.5. The rbinom R function generates a 0 or 1 status according to the predefined probability, and 0 is assigned to the −1. The proportion of samples having CNAs in the total sample was selected among the predefined values (0.1, 0.2, 0.3, 0.4 and 0.5) for each simulated CNA.

After the generation of CNAs, the GEs matrix with similarity with the CNA matrix was simulated. The random values from the normal distribution with different standard deviation (SD) values were added to a simulated CNA for the generation of GEs having various similarities according to the SD values. To simulate a GE matrix having less similarity with the CNA matrix, a greater SD value was applied in the generation of random numbers.

Power analysis was also performed with the simulation data. First, two random CNAs–GEs pairs were generated. The CNA matrices were generated by the same method used in similarity analysis. Then, a random expression matrix was generated and the same matrix was used as an expression matrix in both conditions. The random expression matrix was generated by random numbers from standard normal distribution. Since the CNA matrices were different and the expression matrices were the same between conditions, this generated the true differential interaction of CNAs and GEs between conditions. Simulation data were generated with different parameters, including the number of samples and genes.

### 2.3. Analysis of TCGA-Multiomics Data

In addition to the simulation study, to test whether the current approach identifies valid biological phenomena, TCGA-LGG data were analyzed. The data were downloaded from the genomic data commons (GDC) portal (https://portal.gdc.cancer.gov/), and clinical information was also obtained from the portal. For the detection of CNAs and GEs, Affymetrix 6.0 SNP microarray and Illumina Hiseq 2500 sequencing platform were used, respectively.

For the set-wise CNA expression interaction analysis of the TCGA-LGG data, biological pathway information was used. The current analysis framework can be applied straightforwardly to gene sets that are constructed with the other biological knowledge, such as gene ontology. The pathway information, which is mainly compiled from the Bio-Carta (www.biocarta.com), KEGG (www.genome.jp/kegg) and the Reactome (www.reactome.org) websites, was downloaded from MSigDB of the Broad Institute (https://www.gsea-msigdb.org/gsea/msigdb/index.jsp).

### 2.4. Comparison with Single Gene-Wise CNA Expression Analysis

One of the strengths of the gene set-wise analysis was that it could identify slight changes in genomic signals [28]. Maybe the strength came from the modeling of the interaction between the elements of the gene sets. To find out whether the current set-wise approach had the same advantage, the detection of significant changes in the CNAs and gene expression profiles was performed single gene-wisely. However, previous methods are not implemented to model the difference in interaction between conditions. Therefore, applicable methods for testing the differential change in the interaction of CNAs and GEs between conditions were applied. First, correlation-based single CNA and GE analysis was performed (See Supplementary Methods), and Mantel statistics with different distance measures, including Euclidean, Manhattan and Mahalanobis distances, that were available to the differential interaction analysis, were applied for comparison with Renyi’s relative entropy and Mantel statistics in the analysis of gdCoxS.

## 3. Results

### 3.1. Simulation Analysis Results

**Figure 2:**
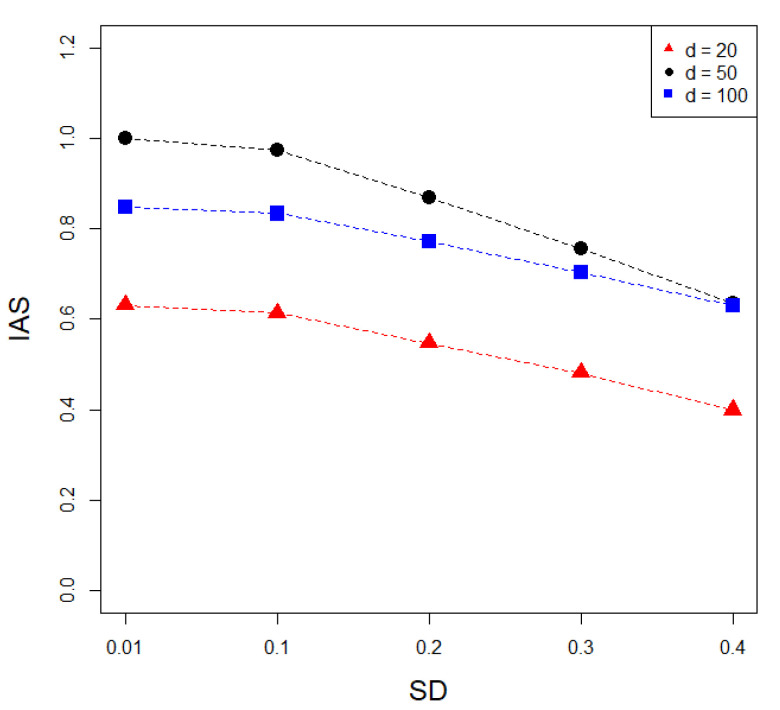
Results of simulation study for measuring similarity between copy number alterations (CNAs) and gene expressions (GEs). The red, black and blue dots and lines indicate the numbers of variables in the gene expression matrix, as 20, 50, and 100, respectively. As standard deviations (SDs) increase, the mean IASs decline.

To generate simulation data for testing whether IAS represents similarity between CNAs and GEs, CNA matrices that have 20, 50, and 100 variables, and 100 samples, were generated. For each simulated CNA, the proportion of the CNA in the total samples was randomly selected from among the predefined frequencies as described in the methods. When a CNA matrix was generated, random values from the normal distribution with SD = 0.01 were added, which resulted in high IAS between the CNA and GE matrices. The second GE matrix was generated by adding random values from normal distribution with SD = 0.1 to the previously generated GE matrix. Likewise, the *i*-th GE matrix was generated by adding random values from normal distribution with SD = (*i* − 1) × 0.1 to the (*i* − 1)-th GE matrix. This generated GE matrices that were less similar to the simulated CNA matrix compared with the previously generated matrix. For each simulated CNA matrix, five GE matrices were generated in total, and this process was iterated 1000 times. When the number of variables in a GE matrix was 100, the same CNA vectors were repeatedly sampled and used for the generation of the GE matrix. Figure 2 shows that the IAS represents the similarity between CNAs and GEs. Each point indicates the mean IAS between the CNA matrices and the simulated expression matrices, with corresponding SD values. In general, the mean IASs were highest when SD was 0.01, and they became lower with increasing SD. The mean IAS was lowest with SD = 0.4 in all simulations. Besides mean values, the paired t tests of the IASs were highly significant between IASs from different SDs (*p* < 2.2 × 10^−^^16^). These indicate that the IAS represents similarity between CNAs and GEs. Since the CNA and GE matrices are different types of data, the simulated matrices should have different distributions. While it was obvious that the simulated CNA matrices have binomial distributions, it was not clear that the simulated GE matrices have multivariate normal distributions that are frequently used in the simulation of a gene expression matrix, because they were generated by adding numbers from binomial and normal distributions. Therefore, normality tests were applied to the GE matrices and the result showed that the matrices had multivariate normal distributions with Bonferroni’s multiple testing correction (data not shown).

**Figure 3:**
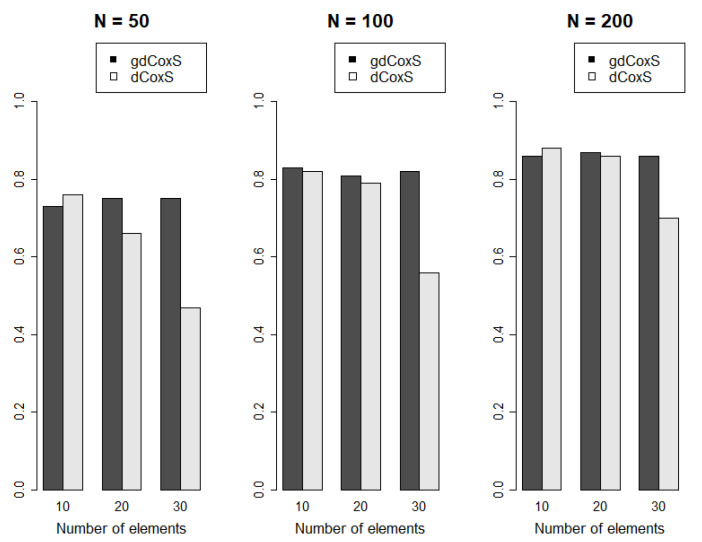
Results of power analysis. The number of x axis is the number of variables in the gene sets, and the y axis represents power. When the number of samples is higher, the overall powers of gdCoxS are higher than the powers with lower number of samples, regardless of the number of variables. The dCoxS shows, however, an obvious trend of decreasing power with elevating numbers of elements of gene sets. N; number of simulation samples in each class.

Power analysis was performed with changes in the number of samples and number of elements in the simulation data. The number of samples included {100, 200, 400}, and the number of variables in the set were set within {10, 20, 30}. The number of permutations was set to 100. Figure 3 shows the results of the power analysis. There was an obvious trend whereby the power of gdCoxS and dCoxS increased as the number of samples was elevated. However, dCoxS had a decreasing power as the number of elements in the gene sets increased, regardless of the number of samples, while dCoxS showed the best performance with the smallest number of elements (*n* = 10). Since the gdCoxS used a covariance matrix for estimating the relationship between variables, gdCoxS captured the difference in CNA matrices more efficiently than the dCoxS, which adopted the productive kernel in estimating density without the use of such a covariance matrix, which was more evident in the higher number of elements in the gene set. Considering the high-dimensional characteristics of functional genomics data, the gdCoxS is a more efficient and robust method, which can detect the dynamics between matrices from two different sets of genomic data.

### 3.2. Real Data Analysis

In the TCGA-LGG, genes of CNA and expression data were mapped to the ensemble identifier system. Since the pathway information used gene symbols, the mapping table of the HUGO Gene Nomenclature Committee (HGNC) for gene symbols and ensemble identifiers was used for mapping gene symbols to ensemble identifier (https://www.genenames.org/download/cus-tom/). The CNA data had 533 samples and the RNA sequencing data had 530 samples. Of the samples, 507 samples with CNA, gene expression and survival information were used in the analysis. In the MSigDB, there were 1335 canonical pathways from the open databases including the KEGG, BioCarta and Reactome. The class was labeled into two groups according to the survival status (death = 98, survival more than 5 years = 409). In the analysis of the TCGA-LGG dataset, the GDC provided CNA information that had been computed using the Genomic Identification of Significant Targets in Cancer (GISITC) algorithm [17]. The CNA information of 12,117 ensemble genes, that were matched to the genes of the 1335 items of MSigDB pathway information, were applied in this analysis. The RNA sequencing (RNA-seq) data has 60,483 transcripts in total, and 13,339 transcripts were mapped to the ensemble identifiers of all the pathway information in the 1335 pathways. Since the RNA-seq data had different batches, a batch effect adjustment was performed with Combat-seq program [29]. After the adjustment, the RNA-seq data were normalized using the quantile normalization method. First, zero values were treated as missing values and they were imputed using the impute R package with default parameters [30]. The data were then log-transformed and the quantile normalization was applied. For the quantile normalization, the normalize.quantiles function of the preprocessCore R package was used [31]. In the real data analysis, pathway gene sets having more than 10 elements were arbitrarily selected for analysis. In total, 1282 pathways were applied for this analysis. The numbers of CNA ensemble identifiers of each pathway ranged from 10 to 933 (median = 23). Those of the pathway expression matrices lay between 10 and 941 (median = 23).

For each pathway, CNA and expression matrices with elements of the pathway were generated, and the differential interaction of two matrices between the survival and death group was computed. To test the significance of the difference of IASs between conditions, a permutation test was applied with 26,000 repeats of permutation. 

In the gdCoxS analysis, Bonferroni’s multiple testing correction was applied (adjusted *p* value = 3.9 × 10^−5^). With the threshold, 577 pathways were found to exhibit significantly different interactions of CNAs and expressions of the pathways between the survival and death groups of TCGA-LGG patients (Table 1 and Supplementary Table S1).

**Figure 4:**
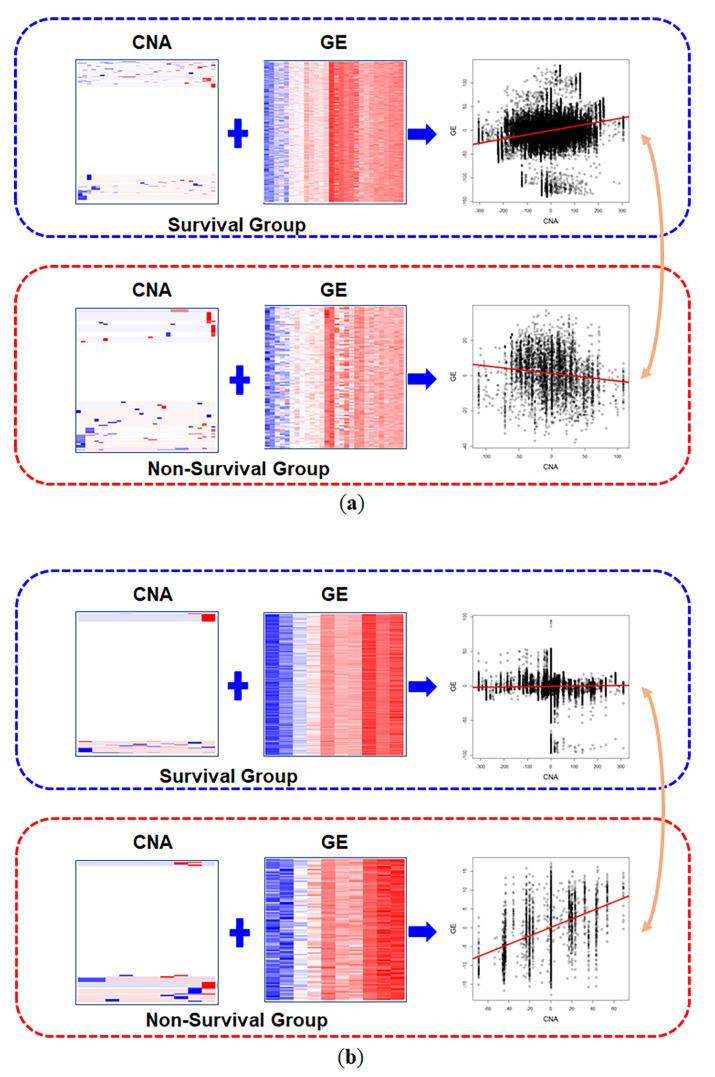
Heatmap and scatter plot of sample-wise distances of pathway copy number alteration and gene expression matrices of the significant results. Note that pathway CNA matrices contain substantial portions of neutral status. The orders of genes in the CNA and GE matrices are set to the same in the survival and non-survival groups. (**a**) Results of ‘GLYCOSPHINGOLIPID_METABOLISM’. (**b**) Results of ‘IL3_PATHWAY’ pathway gene set. The scatter plots are made up of plotting sample-wise distances from CNA and GE matrices. The slopes of red lines in the scatter plots indicate interaction scores of each condition.

In the result, 274 pathways showed increased interactions of CNAs and GEs in the non-survival group, which indicated that variations in CNAs and GEs were more harmonized. On the other hand, 303 pathways had decreased interactions in the non-survival group. The IAS of the IL3_PATHWAY from the pathway interaction database (PID) increased from 0.023 in the survival group to 0.407 in the non-survival group, which was the greatest absolute diffIAS among the results (Figure 4). The ‘GLYCOSPHINGOLIPID_METABOLISM’ pathway from the REACTOME database had the greatest positive diffIAS (= 50.66), which implied that the coordination of the CNAs and GEs of the pathway in the survival group was disrupted in the non-survival group. While the IAS of the pathway CNAs and GEs was 0.256 in the survival group, it decreased (−0.136) in the non-survival group (Figure 4).

For the benchmark analysis of gdCoxS, differential co-expression analysis and Mantel statistics were applied. The differential coexpression analysis includes an estimation of the correlation coefficient between a CNA and GE in each condition, and a test of the significance of the difference in correlations between conditions (See Supplementary Methods). In the single gene-wise differential coexpression analysis, cis and trans regulation were considered, and only the CNAs and GEs that were used in the pathway analysis were included to avoid the loss of power that resulted from a large number of statistical tests. First, 6202 CNAs and 6233 GEs were selected and correlations between the CNAs and GEs were computed in each condition, and the differences in the correlations were tested (Supplementary Methods). After the Bonferroni’s multiple testing correction, there was no significant result from the Bonferroni’s multiple testing correction (adjusted *p* < 1.29 × 10^−9^).

In the benchmark analysis, the Mantel statistics were also applied to compare the performance of gdCoxS when different similarity measures other than Renyi’s relative entropy were applied (Supplementary Methods). Different statistics, including Euclidean, Manhattan and Mahalanobis, which could compute interactions between CNAs and GEs, were applied. Although the Mantel test with different measures showed substantial numbers of significant results, the numbers were far less than those of the gdCoxS analysis (Supplementary Tables S2–S4, respectively). In the result, the Mantel statistics with the Mahalanobis distance using the covariance matrix showed the largest number of significant results (*n* = 171). 

## 4. Discussion

In this research, the gdCoxS performs an integrative analysis of CNAs and GEs. In the simulation analysis, the gdCoxS shows an improvement in the performance in terms of power, especially with larger numbers of gene set elements. In the real data analysis, the gdCoxS detected 577 significant results, while the single gene-wise differential coexpression analysis gave no significant result, and the set-wise analysis with Mantel statistics identified fewer significant pathways than gdCoxS. These results seem to indicate that the gdCoxS outperforms the other benchmark methods.

When the single gene differential coexpression analysis was applied, no significant results could be found in the result of the single gene-wise analysis. However, gene set methods including gdCoxS and Mantel tests identified a lot of significant pathway CNA–GE set pairs. These findings clearly indicate the benefit of gene set-wise analysis, which has more power to detect significant interactions between CNAs and GEs. In the benchmark study using Mantel statistics, the results with Mahalanobis distance showed a far better performance than the other measures. This seems to result from the fact that the Mahalanobis distance uses a covariance matrix that can capture the relationship between elements of gene sets. This finding supports the validity of the concept in gdCoxS, which is an application of the multivariate density function with covariance information to capture the relationship between CNAs explicitly. The dCoxS method was not compared in the real data analysis because variations in sample-wise distances in CNA matrices tended to be zero, which made the computation of IAS intractable. Among the pathways, more than a thousand of pathway CNA matrices showed such variations. This finding strongly indicates that the productive kernel of the dCoxS was not suitable for detecting combinatorial variations in CNAs. In the benchmark analysis, the set-wise methods, such as modified canonical correlation analysis (CCA), that were presented in the introduction could be applied. However, the methods can estimate the similarity between CNA and GE matrices only, and the differences in the similarities between conditions were not considered. Moreover, the methods provided no statistical testing for the estimation of *P* values. Therefore, the comparison between the gdCoxS and the modified CCA was not possible.

In the result, many pathways were related to the glioma pathophysiology in previous studies. For example, 10 pathways were related to p53, which has impacts on the glioma pathophysiology (Supplementary Table S1). The mutation and inactivation of p53 is related to the proliferation and progression of glioma, invasion, and anti-apoptotic activity [32,33,34,35]. It is possible that copy number alterations in p53-related pathways disrupt the CNAs–GE relationship in the favorable group of LGG. The significant change in IASs between the CNAs and GEs of the p53-related pathways in the non-survival group seems to implicate a disrupted regulatory relationship between CNAs and GEs. Considering the role of p53 in the prognosis of many types of cancers [35], these results indicate the validity of gdCoxS analysis. Among the p53-related pathways, the “53 regulates transcription of caspase activators and caspases” pathway is interesting because the result indicated that the differential interaction of CNAs and GEs in the pathway was associated with the apoptosis that is critical to the survival of cancer genes. There are supportive results to this finding. In the pathway, p53 regulates caspase 10, which is associated with apoptotic signaling in glioblastoma [36], and capase 10 induced cellular death in response to the chemotherapeutic agent, which has a possibility of prolonged survival [37]. In the mouse experiment, the ATM gene was involved in the suppression of glioblastoma by the down-regulation of glioblastoma-associated genes such as the PDGFRA gene [38]. P63, which is another member of the pathway, was revealed to suppress tumor growth by up-regulating caspase 1 expression [39]. These seem to be consistent with the results of the significant differential interaction of CNAs and GEs between survival and non-survival groups.

The EGF pathway also indicated the validity of the analysis result (Table 1). The EGF receptor (EGFR) and its downstream signaling is frequently aberrant in cancers, especially in glioma [40]. EFGR gene amplification and overexpression can be observed in approximately 40% of glioblastoma [41]. Since the EGFR signaling is associated with the apoptosis, proliferation and invasion of cancer cells [42], the EGFR was investigated as a therapeutic target in previous studies [43]. The significant change in the interaction of CNAs and GEs in the EGF pathway between the survival and non-survival groups seems to be further supportive evidence of the fact that the EGF and its receptor have a therapeutic potential. It is notable that the homocysteine pathway (‘DEGRADATION OF CYSTEINE AND HOMOCYSTEINE’ from REACTOME database) was highly ranked in the significant results. It is well known that the homocysteine metabolism is aberrant in cancers, including glioma [43], and the homocysteine level is associated with the death of a human glioblastoma cell line [44]. Moreover, the variant of the methylenetetrahydrofolate reductase was shown to be significantly associated with patient survival [45,46]. Considering these, the interaction between CNAs and GEs in the homocysteine pathway seems to be related to the pathophysiology of the lower-grade glioma.

In conclusion, the set-wise identification of the interaction between CNAs and GEs revealed pathways that are consistent with the molecular pathophysiology of lower-grade glioma, which was not found in single-variable analysis. This gene set method for performing the integrative analysis of multi-omics data will promote the discovery of hidden biologic mechanisms.

## Figures and Tables

**Table 1:** Pathways showing upper and lower top 5 significant results in gdCoxS analysis. The total results are listed in Supplementary Table S1.

Pathway Database	Pathways	*N*_CNA_^1^	*N*_EXP_^2^	IAS.S ^3^	IAS.NS ^4^	diffIAS ^5^
PID	IL3_PATHWAY	10	10	0.023	0.407	−52.037
REACTOME	PROTEIN_METHYLATION	14	14	0.197	0.524	−48.578
REACTOME	DUAL_INCISION_IN_GG_NER	14	14	0.081	0.430	−48.002
BIOCARTA	FORMATION_OF_INCISION_COMPLEX_IN_GG_NER	26	26	0.176	0.501	−47.231
REACTOME	MICRORNA_MIRNA_BIOGENESIS	10	10	0.146	0.476	−47.219
REACTOME	TRIGLYCERIDE_CATABOLISM	15	11	0.148	−0.179	41.893
REACTOME	DEGRADATION_OF_CYSTEINE_AND_HOMOCYSTEINE	11	10	0.168	−0.159	41.940
BIOCARTA	EGF_PATHWAY	14	14	0.200	−0.145	44.284
KEGG	CYTOSOLIC_DNA_SENSING_PATHWAY	16	15	0.240	−0.127	47.355
REACTOME	GLYCOSPHINGOLIPID_METABOLISM	31	28	0.256	−0.136	50.660

^1^*N*_CNA_: number of variables in copy number matrix; ^2^*N*_CNA_: number of variables in gene expression matrix; ^3^ IAS.S: interaction score in survival group; ^4^ IAS.NS: interaction score in non-survival group; ^5^ diffIAS: difference of interaction scores; PID: pathway interaction database; KEGG: Kyoto Encyclopedia of Genes and Genomes.

## Supplementary Materials

The Supplementary Materials are available online at https://www.mdpi.com/1099-4300/22/12/1434/s1.
