# Supplementary material for: Set-Wise Differential Interaction between Copy Number Alterations and Gene Expressions of Lower-Grade Glioma Reveals Prognosis-Associated Pathways
Source: Entropy (Basel). 2020 Dec 18;22(12):1434. doi: 10.3390/e22121434 (PMC7765960; doi:10.3390/e22121434)
Supplement: Supplementary file 1 [file entropy-22-01434-s001.zip › gdcoxs_entropy_supplementary_material.docx]

**Supplementary Methods**

**Permutation of gdCoxS**

The permutation process of gdCoxS is the same as that of dCoxS. When there is a condition 1 (C_1_) and condition 2 (C_2_), interaction score (IAS) was determined at each condition. Then, the IAS values were transformed by Fisher’s transformation, which is

$$Z=\frac{1}{2}\times\left[\frac{\log(1+IAS)}{\log(1-IAS)}\right]$$

And then, different of IAS was determined as follows

$$diffIAS(IAS_{C_{1}},IAS_{C_{2}})=\frac{(Z_{C_{1}}-Z_{C_{2}})}{\sqrt{1/(N_{1}-3)+1/(N_{2}-3)}}$$

Permutation is performed with sample-wise distance matrices as in the test of Mantel statistics.

$$P=\frac{\sum_{i=1}^{N} I(|diffIAS(IAS_{c_{1}},IAS_{c_{2}})|\text{ }<\text{ }|diffIAS(pIAS_{C_{1}},pIAS_{C_{2}})|)}{N}$$

In the above equation, *I()* is an indicator function and *N* is a number of permutation. For each permutation, sample-wise distances were permuted and permuted *IAS* (*pIAS*) was generated in each condition. The *diffIAS* of the permuted *pIASs* was then determined and the number of cases when the *diffIAS* of *pIASs* were greater than the original *diffIAS* was divided by the total number of permutations.

**Single gene-wise differential CNA-expression analysis**

For comparison of performance of gdCoxS, the Fisher’s differential correlation, gdCoxS with Euclidean, Manhattan and Mahalanobis distance was applied.

The Fisher’s method was applied to the meta-analysis of the differentially co-expressed genes (Choi *et al.,* 2005). After computation of correlation coefficient of gene pairs, Fisher’s Z transformation was performed for each gene pair.

$$Zf=\frac{1}{2}\times\ln(\frac{1+CC}{1-CC})$$

In above equation, CC was correlation coefficient of a gene pair in each condition. After transformation, the significance of conditional difference of the Z transformed correlation coefficients were tested using following statistic.

$$P(Z\geq|\frac{(Zf_{1}-Zf_{2})}{\sqrt{1/(N_{1}-3)+1/(N_{2}-3)}}|)$$

*Zf_1_* and *Zf_2_* were the Fisher transformed correlation coefficients in each condition. *N_1_* and *N_2_* were numbers of samples in each condition group. In the Choi’s work, difference of the Z transformed correlation coefficients were determined using effect size method. Since this research was not meta-analysis, the differences of Z-transformed correlation coefficients were tested using standard normal distribution.

For benchmark analysis with Mantel test, three available methods including Euclidean, Manhattan and Mahalanobis distances were applied. The Euclidean distance was measured as follows. When the Si = (Si1, Si2, Si3….. Sik) and Sj = (Sj1, Sj2, Sj3….. Sjk) is ith sample and jth sample of the data matrix having k variables, each distance was determined by following equations.

$$\text{EuclideanDist[}i,j]=\sqrt{\sum_{1}^{k} (S_{ik}-S_{jk})^{2}}$$

$$\text{ManhattanDist[}i,j]=\sqrt{\sum_{1}^{k} \left|S_{ik}-S_{jk}\right|}$$

$$\text{MahalanobisDist[}i,j]=\sqrt{(S_{i}-S_{j})\sum^{-1}(S_{i}-S_{j})}$$

In the Mahalanobis distance, Σ^–1^ is an inverse of covariance matrix of matrix.

**References**

Cho, S.B. *et al.* (2009) Identifying set-wise differential co-expression in gene expression microarray data. *BMC Bioinformatics*, 16(10), 109.

Choi, J.K. *et al*. (2005) Differential coexpression analysis using microarray data and its application to human cancer. *Bioinformatics*, 21(24), 4348-55.
